# Effects of Liraglutide on Left Ventricular Function: A Meta-Analysis of Randomized, Placebo-Controlled Trials

**DOI:** 10.1155/2021/9993229

**Published:** 2021-06-15

**Authors:** Zhaoshuang Zhong, Kaiming Chen, Yan Zhao, Shuyue Xia

**Affiliations:** ^1^Department of Respiratory, Central Hospital, Shenyang Medical College, Shenyang, China; ^2^Department of Cardiovascular Disease, Central Hospital, Shenyang Medical College, Shenyang, China

## Abstract

**Background:**

The effects of liraglutide treatment on the left ventricular systolic and diastolic function remain unclear.

**Methods:**

This meta-analysis was conducted according to the preferred reporting items for systematic reviews and meta-analysis (PRISMA) statement. All relevant randomized, placebo-controlled trials (RCTs) were identified by searching PubMed, EMBASE, Cochrane Library, and ISI Web of Science from the establishment to January 2021 without language limitations. The weighted mean difference (WMD) with 95% confidence intervals (CIs) was calculated.

**Results:**

Ten placebo-controlled RCTs involving a total of 732 cases were included in the meta-analysis. Compared with the placebo group, liraglutide therapy showed no beneficial effect on the left ventricular ejection fraction (LVEF) at the end of the study (WMD: 2.120, 95% CI: −0.688 to 4.929, *P*=0.139) and ΔLVEF during the trial period (WMD: −0.651, 95% CI: −1.649 to 0.348, *P*=0.202). Similarly, no statistical differences were noted in diastolic function parameters between the two groups, including the value early diastolic filling velocity (*E*)/the mitral annular early diastolic velocity (*e*′) (WMD: −0.763, 95% CI: −2.157 to 0.630, *P*=0.283), Δ*e*′ (WMD: −0.069, 95% CI: −0.481 to 0.343, *P*=0.742), and Δ*E*/*e*′ (WMD: −0.683, 95% CI: −1.663 to 0.298, *P*=0.172).

**Conclusions:**

Liraglutide treatment did not improve the left ventricular systolic and diastolic function. Given the study's limitations, further investigation may be warranted.

## 1. Introduction

Glucagon-like peptide-1 (GLP-1) is a gut-derived hormone that can stimulate glucose-dependent insulin secretion from pancreatic beta cells in response to intake [1, 2]. Since GLP-1 is degraded rapidly by the enzyme dipeptidyl peptidase-4 (DPP-4), GLP-1 receptor agonists with natural or modified structures resistant to the inactivation by DPP-4 were developed [3]. Among the GLP-1 receptor agonists, liraglutide was proved to be associated with a lower incidence of cardiovascular disease (CVD) events compared to placebo [4]. The potential mechanisms may involve improving postprandial lipid metabolism [5] and anti-inflammatory effects [6], leading to benefits on other cardiometabolic risk factors [7].

However, the effects of liraglutide on cardiac function remain unclear. In the Liraglutide Effect and Action in Diabetes: Evaluation of Cardiovascular Outcome Results (LEADER) trial [8], liraglutide treatment failed to reach the statistical significance on the endpoint of hospitalization for heart failure. On the contrary, in a recent meta-analysis, GLP-1 receptor agonists did show beneficial effects on heart failure outcomes, though the explanation for the results was still pending [9]. Many other studies showed similar controversial results. To further clarify the effects of liraglutide on left ventricular function, we performed this meta-analysis with randomized, placebo-controlled trials (RCTs).

## 2. Methods

### 2.1. Search Strategy

This meta-analysis was conducted according to the preferred reporting items for systematic reviews and meta-analysis (PRISMA) statement [10]. All published RCTs comparing the effects of liraglutide and placebo on left ventricular function were identified by searching PubMed, EMBASE, Cochrane Library, and ISI Web of Science from the establishment to January 2021 without language restrictions. The search formula was performed as (liraglutide) AND (left ventricular) AND (randomized). Two authors went through the titles and abstracts of the records and identified eligible articles independently. Disagreements were resolved by discussion or referring to the third author (S.-Y.X.).

### 2.2. Inclusion and Exclusion Criteria

The inclusion criteria contained the following: (1) RCT, (2) liraglutide therapy was administered and compared with the placebo-controlled group, and (3) reported at least one of the following outcomes: for systolic function, the left ventricular ejection fraction (LVEF) at the end of the study or the changes of LVEF during the study (ΔLVEF); for diastolic function, the mitral annular early diastolic velocity (*e*′), the value early diastolic filling velocity (*E*)/*e*′, or the changes of the two indicators during the trial (Δ*e*′ and Δ*E*/*e*′). Duplicated publications, reviews, meeting abstracts, and case reports were excluded.

### 2.3. Data Extraction and Quality Assessment

Two authors used a predesigned structured form to extract data from each study independently. The data elements included (1) study information, such as the first author's name, publication year, sample size, intervention strategies, and follow-up information; (2) patient characteristics, such as the mean age, the proportion of hypertension, diabetes, and smokers; (3) measurement of the baseline LVEF, *e*′, and *E*/*e*′; (4) outcomes as listed above. The methodological qualities of the included trials were assessed by two independent authors using the modified Jadad scale [11]. The disagreements were resolved by discussion or referring to the third author (S.-Y.X.).

### 2.4. Statistical Analysis

We calculated the weighted mean difference (WMD) with 95% confidence intervals (CIs) for continuous outcomes. The *I*^2^ statistic was used to measure the heterogeneity across the included studies, and a random-effect (RE) model was applied for all pooled outcomes regardless of heterogeneity [12]. In the case of significant heterogeneity, the sensitivity or subgroup analysis would be considered. The publication bias was evaluated by funnel plots with Begg's test [13]. Two-sided *P* < 0.05 indicated a statistical significance. All analyses were completed using Stata v12.0 (Stata Corp, College Station, TX, USA) with the metan function.

## 3. Results

### 3.1. Basic Characteristics of Included Studies

**Figure 1:**
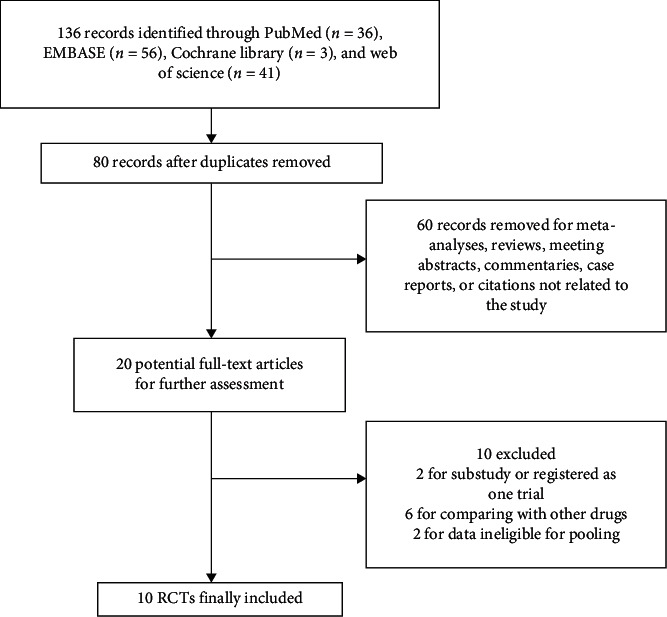
Flowchart of study selection.

A total of 136 records were initially identified by our search strategy, in which 56 duplicates were removed. After a title and abstract screening, another 60 citations were excluded as reviews, meeting abstracts, commentaries, case reports, or irrelevant studies. Among the 20 full-text review articles, ten were further excluded for reasons such as substudy [14], registering as one trial [15], comparing with other drugs, or no available data for pooling [16, 17]. Finally, ten placebo-controlled RCTs [18–27] involving a total of 732 cases were included in the meta-analysis. The detailed flowchart is shown in Figure 1. The baseline characteristics of the studies are presented in Tables 1 and 2. The sensitivity analyses and publication assessments for each endpoint are listed in the Supplementary Materials (online suppl. Figures 1 and 2). No publication bias was found.

### 3.2. Methodological Quality Assessment

The methodological quality was assessed by the modified Jadad scale, composed of randomization, double blinding, withdrawals and dropouts, and allocation concealment [11]. The modified Jadad scores of the enrolled trials ranged from 5 to 7, as presented in Table 3.

### 3.3. Meta-Analysis Results

#### 3.3.1. Left Ventricular Systolic Function

**Figure 2:**
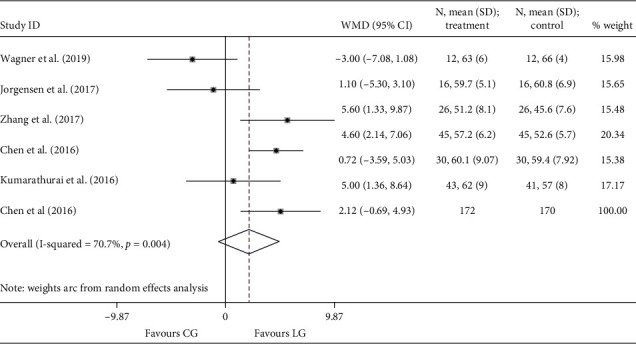
Forest plot for LVEF. LVEF: left ventricular ejection fraction; WMD: weighted mean difference; LG: liraglutide group; CG: control group.

**Figure 3:**
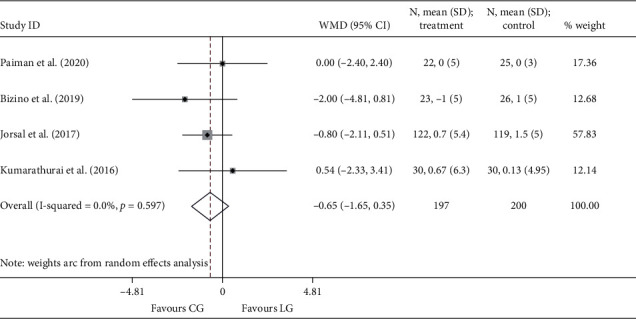
Forest plot for ΔLVEF. LVEF: left ventricular ejection fraction; WMD: weighted mean difference; LG: liraglutide group; CG: control group.

Six studies [20, 22, 23, 25–27] involving 342 cases reported the LVEF at the end of the study. Compared to the placebo group, liraglutide therapy showed no benefits on LVEF (*I*^2^ = 70.7%; WMD: 2.120, 95% CI: −0.688 to 4.929, *P*=0.139) (Figure 2). The outcome of ΔLVEF was reported by four RCTs [19, 21, 24, 26], including 397 cases. No difference was noted between the liraglutide and the placebo group as well (*I*^2^ = 0.0%; WMD: −0.651, 95% CI: −1.649 to 0.348, *P*=0.202) (Figure 3).

#### 3.3.2. Left Ventricular Diastolic Function

**Figure 4:**
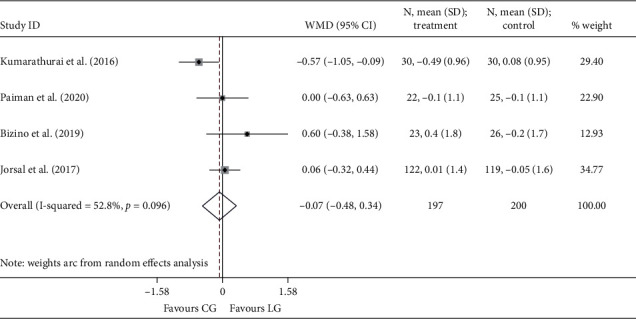
Forest plot forΔ*e*′. *E*′: the mitral annular early diastolic velocity; WMD: weighted mean difference; LG: liraglutide group; CG: control group.

**Figure 5:**
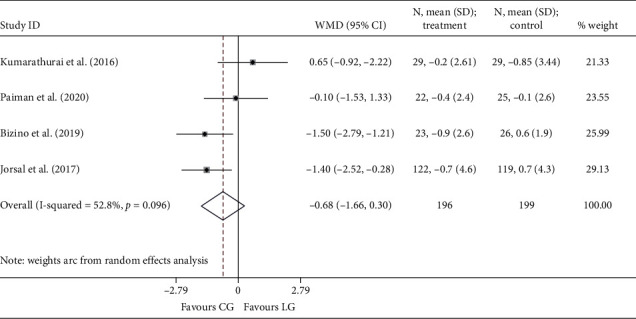
Forest plot forΔ*E*/*e*′. *E*: the value early diastolic filling velocity; *e*′: the mitral annular early diastolic velocity; WMD: weighted mean difference; LG: liraglutide group; CG: control group.

Since only one study [18] reported the endpoint of *e*′, no data were available for the pooling analysis. Three RCTs [18, 20, 22] presented *E*/*e*′ at the end of the study, and the results were comparable between the liraglutide and the placebo group (*I*^2^ = 0.0%; WMD: −0.763, 95% CI: −2.157 to 0.630, *P*=0.283). Four studies [18, 19, 21, 24] reported the outcomes of Δ*e*′ and Δ*E*/*e*′. Similarly, no statistical differences were noted between the two groups, neither Δ*e*′ (*I*^2^ = 52.8%; WMD: −0.069, 95% CI: −0.481 to 0.343, *P*=0.742) (Figure 4) nor Δ*E*/*e*′ (*I*^2^ = 53.2%; WMD: −0.683, 95% CI: −1.663 to 0.298, *P*=0.172) (Figure 5).

## 4. Discussion

In this meta-analysis of placebo-controlled RCTs, we examined the effect of liraglutide treatment on left ventricular function, with or without diabetes, heart failure, and coronary artery disease. The parameters of LVEF, *e*′, and *E*/*e*′ were measured by echocardiography, magnetic resonance, or the pulse indicator continuous cardiac output (PICCO) system. The results showed that liraglutide did not affect the left ventricular systolic or diastolic function.

GLP-1 receptor agonists demonstrated beneficial effects on CVD events and mortality in several studies [28]. The potential mechanisms may involve improving postprandial lipid metabolism [5], endothelial function and anti-inflammatory effects [29, 30], and other benefits directly on the coronary blood flow and myocardial energy metabolism [31–33]. However, the effects of GLP-1 receptor agonists on left ventricular function remain to be established. In previous studies, GLP-1 was associated with improved left ventricular systolic function [34–36]. As a primary GLP-1 receptor agonist, liraglutide showed beneficial effects on LVEF as well, even in patients without diabetes [25, 37]. Nevertheless, most of the studies' population was relatively small or not placebo-matched. In the LEADER trial, liraglutide showed no effect on hospitalization for heart failure, and many other studies showed similar controversial results [8].

To clarify this issue, we conducted the present study, and the results showed that liraglutide had no positive effects on LVEF. In our study, the outcome of LVEF at the end of the study showed significant heterogeneity (*I*^2^ = 70.7%). We performed the sensitivity analysis, and the results are listed in the Supplementary Materials (online suppl.  Figure 1). Due to the limited number of enrolled studies, the subgroup analysis was not carried out. Given the influence on statistical power, we would prefer to regard LVEF at the end of the study as an ancillary finding to ΔLVEF. Furthermore, though not included in the meta-analysis due to the data format, the Functional Impact of GLP-1 for Heart Failure Treatment (FIGHT) trial also showed comparable results between liraglutide and placebo on left ventricular systolic function [16]. Consequently, as hypothesized by some scholars, the beneficial effect of GLP-1 receptor agonists on hospital admission for heart failure may attribute to the reduction in myocardial infarction rather than immediate improvement in systolic function [9].

The effects of liraglutide on left ventricular diastolic function were also investigated. Lambadiari et al. reported that a six-month treatment with liraglutide improves arterial stiffness and myocardial relaxation [38]. Bizino et al. found a 6-month treatment with liraglutide reduced early left ventricular diastolic filling and filling pressure [21]. On the contrary, Nyström et al. reported that, after an 18-week treatment of liraglutide, no improvement of diastolic function was observed compared with glimepiride [39]. In the study conducted by Jorgensen et al., liraglutide may weaken the beneficial effects of exercise on left ventricular diastolic function [22]. The present meta-analysis further confirmed that liraglutide had no benefits on left ventricular diastolic function. The potential explanations may be multiple. Several studies reported that GLP-1 receptor agonists, including liraglutide, were associated with a significant increase in heart rate [40, 41], which means adverse effects on left ventricular function [42] and cardiovascular mortality [43]. The mechanisms may involve sympathetic activity stimulation [44] and immediate effect on GLP-1 receptors in the sinoatrial node [45]. It was hypothesized that beta blocker or other heart rate-lowering drugs might blunt this potential adverse effect [18]. However, in the LIVE study, an increase in heart rate was still observed in patients treating with maximum tolerable beta-blocker dose [24].

As far as we know, the present study is the first meta-analysis assessing the effect of liraglutide treatment on left ventricular systolic and diastolic function parameters using placebo-controlled RCTs. However, the potential limitations of the study should not be ignored. Due to the limited study number and population size, the power of the funnel plot asymmetry test for publication bias might be restricted, and we did not perform the subgroup analyses based on patients with or without diabetes, heart failure, and coronary disease, which might be responsible for the inconsistent findings across the enrolled studies. Furthermore, though high-quality RCTs were enrolled, few data were available for pooling analysis, such as cardiac index, global longitudinal strain, and *E*/*A* ratio. Despite this, LVEF, *e*′, and *E*/*e*′ were the most representative indicators for left ventricular function assessment, and the change in *E*/*e*′ was associated with the NT-proBNP level [14]. Finally, the meta-analysis contained studies with different follow-up periods, intervention regimens, and patient's clinical features, which may also affect the analysis power. Therefore, the study results should be interpreted with caution and may warrant further investigation.

## 5. Conclusion

The present study demonstrated that liraglutide treatment did not improve left ventricular function, irrespective of systolic or diastolic parameters. Given the study's limitations, the results should be interpreted cautiously, and further investigation may be needed.

## Figures and Tables

**Table 1:** Characteristics of included studies.

Study	Year	LG/CG	Sample size, *n*	Age, *y*	Males, *n* (%)	Hypertension, *n* (%)	Diabetes, *n* (%)	Smokers, *n* (%)	Follow-up
Kumarathurai et al. [18]	2021	LG	30	63.1 ± 6.6	24 (80)	23 (77)	30 (100)	14 (36)	12 weeks
		CG	30						

Paiman et al. [19]	2020	LG	22	55 ± 11	8 (36)	NR	22 (100)	8 (36)	26 weeks
		CG	25	55 ± 9	11 (44)		25 (100)	5 (20)	

Wagner et al. [20]	2019	LG	12	53.2 ± 9.7	4 (33)	7 (58)	12 (100)	2 (17)	6 months
		CG	12	52.6 ± 13.8	5 (42)	8 (67)	12 (100)	3 (25)	

Bizino et al. [21]	2019	LG	23	60 ± 6	14 (61)	NR	23 (100)	13 (56)	26 weeks
		CG	26	59 ± 7	15 (58)		26 (100)	18 (69)	

Jorgensen et al. [22]	2017	LG	16	57 ± 10	NR	NR	16 (100)	NR	16 weeks
		CG	16				16 (100)		

Zhang et al. [23]	2017	LG	26	59.1 ± 11.8	20 (77)	17 (65)	5 (19)	15 (58)	1 week
		CG	26	58.7 ± 11.4	19 (73)	16 (62)	7 (27)	17 (65)	

Jorsal et al. [24]	2017	LG	122	65 ± 9.2	109 (89)	NR	39 (32)	25 (21)	24 weeks
		CG	119	65 ± 10.7	106 (89)		35 (29)	23 (19)	

Chen et al. [25]	2016	LG	45	58.0 ± 11.7	34 (76)	27 (60)	9 (20)	25 (56)	3 months
		CG	45	59.0 ± 12.1	32 (71)	29 (64)	13 (28)	27 (60)	

Kumarathurai et al. [26]	2016	LG	30	61.8 ± 7.6	31 (79)	29 (74)	30 (100)	14 (36)	12 weeks
		CG	30						

Chen et al. [27]	2016	LG	39	57.1 ± 13.0	27 (69)	18 (46)	5 (13)	13 (33)	3 months
		CG	38	58.7 ± 12.7	26 (68)	19 (48)	7 (18)	14 (37)	

LG: liraglutide group; CG: control group; NR: not reported.

**Table 2:** Characteristics of included studies.

Study	Year	Liraglutide intervention	LG/CG	LVEF (%)	*e*′ (cm/s)	*E*/*e*′	Heart rate (bpm)
Kumarathurai et al. [18]	2021	0.6 mg/day, increased every 14 days up to 1.8 mg/day	LG	58.9 ± 7.2	5.7 ± 1.6	15.36 ± 6.4	68.6 ± 10.1
			CG				

Paiman et al. [19]	2020	0.6 mg/day, increased every 7 days up to 1.8 mg/day	LG	56 ± 8	5.3 ± 2.1	7.4 ± 3.9	73 ± 13
			CG	57 ± 7	5.7 ± 1.9	7.4 ± 3.3	77 ± 11

Wagner et al. [20]	2019	0.6 mg/day, increased every 7 days up to 1.8 mg/day	LG	62 ± 7	NR	8.8 ± 2.3	NR
			CG	64 ± 5		9.7 ± 2.6	

Bizino et al. [21]	2019	0.6 mg/day, increased every 7 days up to 1.8 mg/day	LG	55 ± 5.8	6.0 ± 1.6	7.3 ± 2.9	70 ± 9
			CG	55 ± 4.5	6.0 ± 1.8	7.9 ± 2.3	70 ± 12

Jorgensen et al. [22]	2017	0.6 mg/day, increased every 7 days up to 1.8 mg/day	LG	59.2 ± 6.1	NR	8.1 ± 1.9	80.4 ± 8.5
			CG	60.7 ± 6.6		8.2 ± 2.3	81.3 ± 8.3

Zhang et al. [23]	2017	0.6 mg/day for 2 days, 1.2 mg/day for 2 days, 1.8 mg/day for 3 days	LG	42.2 ± 7.1	NR	NR	67 ± 10
			CG	42.1 ± 7.3			67 ± 11

Jorsal et al. [24]	2017	0.6 mg/day, increased every 7 days up to 1.8 mg/day	LG	33.7 ± 7.6	6.6 ± 2.1	12.6 ± 6.0	76.3 ± 15.1 75.1 ± 9.6
			CG	35.4 ± 9.4	6.9 ± 2.4	11.7 ± 5.5	

Chen et al. [25]	2016	0.6 mg/day for 2 days, 1.2 mg/day for 2 days, 1.8 mg/day for 3 days	LG	47.2 ± 5.1	NR	NR	71.7 ± 12.1
			CG	47.7 ± 5.1			

Kumarathurai et al. [26]	2016	0.6 mg/day, increased every 14 days up to 1.8 mg/day	LG	58.9 ± 7.6	NR	NR	NR
			CG				

Chen et al. [27]	2016	1.8 mg before intervention, 0.6 mg/day for 2 days, 1.2 mg/day for 2 days, 1.8 mg/day for 3 days	LG	51.3 ± 8.1	NR	NR	NR
			CG	50.7 ± 7.6			

LG: liraglutide group; CG: control group; NR: not reported; LVEF: left ventricular ejection fraction; *E*: the value early diastolic filling velocity; *e*′: the mitral annular early diastolic velocity.

**Table 3:** Assessment of the methodological quality of included studies [11].

Author	Randomization	Double blinding	Allocation concealment	Withdrawals/dropouts	Scores
Kumarathurai et al. [18]	Yes	Yes	Unclear	Yes	6
Paiman et al. [19]	Yes	Yes	Yes	Yes	7
Wagner et al. [20]	Yes	Yes	Unclear	Yes	6
Bizino et al. [21]	Yes	Yes	Yes	Yes	7
Jorgensen et al. [22]	Yes (method unclear)	Yes	Unclear	Yes	5
Zhang et al. [23]	Yes	Yes	Unclear	Yes	6
Jorsal et al. [24]	Yes	Yes	Yes	Yes	7
Chen et al. [25]	Yes	Yes	Unclear	Yes	6
Kumarathurai et al. [26]	Yes	Yes	Unclear	Yes	6
Chen et al. [27]	Yes	Yes	Unclear	Yes	6

## Data Availability

All the data that support the findings of this study are included within the article.

## Supplementary Materials

Supplementary Materials Online suppl. Figure 1: sensitivity analysis for the endpoint of A, LVEF; B, ΔLVEF; C, *E*/*e*′; D, Δ*e*′; E, Δ*E*/*e*′. Online suppl. Figure 2: funnel plot for the endpoint of A, LVEF; B, ΔLVEF; C, *E*/*e*′; D, Δ*e*′; E, Δ*E*/*e*′.
