# Supplementary material for: Effects of Liraglutide on Left Ventricular Function: A Meta-Analysis of Randomized, Placebo-Controlled Trials
Source: Int J Endocrinol. 2021 Jun 15;2021:9993229. doi: 10.1155/2021/9993229 (PMC8219465; doi:10.1155/2021/9993229)
Supplement: Supplementary Materials — Online suppl. Figure 1: sensitivity analysis for the endpoint of A, LVEF; B, ΔLVEF; C, E/e′; D, Δe′; E, ΔE/e′. Online suppl. Figure 2: funnel plot for the endpoint of A, LVEF; B, ΔLVEF; C, E/e′; D, Δe′; E, ΔE/e′. [file 9993229.f1.zip › 9993229.f1/Online suppl. Fig 1.doc]

Online suppl. Fig 1 Sensitivity analysis for endpoint of A LVEF; B ΔLVEF; C E/e’; D Δe’; E ΔE/e’. LVEF= left ventricular ejection fraction; E= the value early diastolic filling velocity; e’= the mitral annular early diastolic velocity.
